# Southern Versus Northern Practice

**Published:** 1848-10

**Authors:** B. Rush Mitchell

**Affiliations:** U. S. Navy


### MEDICAL EXAMINER,

AND

### RECORD OF MEDICAL SCIENCE.

NEW SERIES.—NO. XLVI.—OCTOBER, 1848.

### ORIGINAL COMMUNICATIONS.

*Southern* versus *Northern Practice.* By B. Rush Mitchell, M. D., U. S. Navy.

Within a few years past, and more especially since the multiplication of Medical Schools in the South and West seemed to require its advocacy, the doctrine has been held, and diligently impressed upon the public mind, that there was a difference between Northern and Southern practice, so well defined and determined, that he who should attempt to treat disease in the South according to Northern dicta, must be almost uniformly unsuccessful; and so frequently have Southern writers and teachers asserted this dogma, so animated have they been in arguing its truth, that they have often, we doubt not, become convinced of that which was before to them only a speculative opinion.

Without pausing to notice the fact, that the first enunciation of the proposition we are considering was under the auspices of a Western Medical School, and urged as a reason for the attendance of medical students to its teachings, in preference to those of Eastern contemporaries; and that a majority of the teachers who supported the proposition were, themselves, the *alumni* of Eastern and Northern Medical Schools, it is sufficient for us to know, that the belief adverted to is now, and has been for some years, a predominant article of the medical creed held by Southern and Western physicians. In truth, we ourselves, until within a few months past, have been accustomed to regard a graduate of a Northern or Eastern School as entirely incompetent to battle with and master Southern diseases; nor was it until the reverse became matter of demonstration, that we could believe otherwise. While, however, we repudiate in toto the slanders upon Northern physicians, it must be confessed that between the diseases of the Northern and Southern sections of our Union there are differences well marked and apparent. But that these differences, of themselves, constitute, as has been alleged, any reason why a well educated medical man from a Northern city cannot master them until he re-study his profession, is, in our judgment, wholly erroneous. This will be evident from a consideration of the differences themselves. These arrange themselves under four heads. First, the suddenness and intensity of invasion; secondly, the inaptitude of reaction; thirdly, the rapidity with which fatal or healthful terminations ensue; fourthly, the increased quantity of medicine not only tolerated but required. An attentive examination of these points of difference will, we think, at once demonstrate how groundless is the assumption of our Western and Southern medical friends. We grant that there *was* a time when their proposition might have been true; in those days, when physicians, versed in books only, and anxious only to name a disease that they might find from the books its treatment, were lost in a wilderness of conjecture when conflicting and strange symptoms were present; but at this day, when medical men rely upon principles in medicine and their multifarious application in the treatment of disease, it would seem strange that transference of residence should strip their principles of utility, or themselves of skill.

We hold, and justly, that if medicine be a science, the principles which it furnishes must be of universal application. Either a medical principle which obtains in the North must be equally valid in the South, or it is no principle at all. Principles recognize no degrees of latitude, no varieties of climate in their application; and he who understands both their nature and teaching aright enjoys an equally wide field of practice. To one well versed in the principles of medicine, it matters little whether the disease he is treating be under a snowy or a tropical sky; or by what name it may be called. These are matters of minor importance. The grand questions he has to solve are,—what diseased manifestations are present ; what implication of organs do they indicate ; and having determined these, his principles will guide him to the correct practice.

Examine in this light the differences which we have enumerated as existing between Northern and Southern diseases ; and it will hardly be pretended that they constitute stumbling blocks to a *thoroughly* educated physician. Is a disease sudden and intense in its invasion, — he can ascertain that as easily as another, although he see the disease for the first time. Does reaction fail to come on,—he can perceive it as well as others. Does reaction come on slowly,—he can detect that also, and expedite its access. Are the tendencies of the disease to a speedily fatal or heathful issue, — these tendencies are not voiceless, and they will speak equally audibly to a Northern as to a Southern ear. In fact, all these differences are entirely *sensual*, their evidence is addressed to the eye ; and any medical man who can interpret signs and symptoms, can ascertain their indications, although he, a native of Greenland, were to find them in Louisiana.

The fourth difference, viz., in the quantity of medicine required, is the only one which appears to possess any element of deception or misdirection to the Northern physician. We say *appears*, for it really possesses nothing of the kind. It is self-evident that, if the physician has duly and attentively remarked the nature of the differences we have before noticed, they will, of themselves, indicate the necessity of a more energetic practice than that which he has been wont to employ. If he be a well educated physician, the sudden and violent onset of the disease, the want of reaction, and a marked tendency to a speedily fatal issue of the case before him, will indicate to his reason far more authoritatively than any other voice can speak, the necessity for prompt, energetic and decisive treatment. He decides, therefore, that a certain end must be attained within a given time ; he is satisfied that a certain remedy alone will answer his purpose ; and having arrived at these conclusions, *dose* becomes matter of small regard—effect is the object sought. That he may not carry his remedy far enough in his first case is readily admitted, but that he will fail in this particular in the second, is hardly probable.

In view of these reasons, then, we conclude that the proposition of our Southern brethren is utterly untenable, as far as thoroughly instructed Northern physicians are concerned :—we say thoroughly instructed physicians, because we apprehend that the ignorance of many upon whom the title of Doctor has been conferred of late years has been the grand cause for our Southern friends so misjudging Northern medical men. So vast and so motley has been the concourse of aspirants for medical honours during the last ten years, and so numerous the rival schools of medicine, that it were indeed marvellous if, in the struggle of both, a great number of unworthy individuals have not been invested with the doctorate, nor that the standard of medical education being lowered, even the really deserving and studious have failed to be as well grounded as they should have been, in the principles of their professsion.

It is not then to these, and such as these, that our Southern friends should point to establish their opinion. We doubt not that many of these have to re-learn, or rather begin to learn, ere they can practice successfully at the South; for so they would have to do anywhere. Let not our Southern brethren judge from such examples. Let them, however, inquire where the most successful practitioners of the tropics were educated ; at what colleges the practitioners of India and the Isles derived that knowledge which has rendered them more successful than native born physicians. Let them ascertain in what section of the country the medical officers of the Navy were educated ; and how it happens that they succeed so well in every clime. And let them say, too, why it was, that during the prevalence of epidemic yellow fever in the Gulf of Mexico last summer, when the treatment of the disease was in the hands of junior officers, most of whom had never seen a case, the Navy should have lost but *one in thirty* cases ; whilst at Vera Cruz, in the army hospitals, where the sick were attended by veteran yellow fever practitioners, who were specially and for that reason appointed to that duty, the ratio of deaths was about *one in four*. These junior officers were the alumni of Northern schools ; the cases they treated were in unacclimated subjects ; the disease they knew only by description, yet they were thus successful. And if medical practice educed from medical principles was thus successful in yellow fever, we have a right to presume that it would not fail in other Southern diseases.

We conclude, then, that disease is an inroad upon life, varying in its mode of access, and in its expression, according to climatorial, local, or individual peculiarities; and that the physician who rightly understands and interprets its general history, will never be at a loss to appreciate its minor details or peculiarities, nor to address to each its appropriate means of removal. No one incapable of doing this merits the name of a physician; while to be fully competent to the task we believe to be within the grasp of every anxious, patient, and thoughtful student of medicine. Thus viewed, medicine is a world-wide science, and whether her assistance be invoked North or South will be prompt, in scientific hands, to direct a proper treatment.

